# WGCNA: an R package for weighted correlation network analysis

**DOI:** 10.1186/1471-2105-9-559

**Published:** 2008-12-29

**Authors:** Peter Langfelder, Steve Horvath

**Affiliations:** 1 Department of Human Genetics, University of California, Los Angeles, CA 90095, USA; 2 Department of Human Genetics and Department of Biostatistics, University of California, Los Angeles, CA 90095, USA

## Abstract

**Background:**

Correlation networks are increasingly being used in bioinformatics applications. For example, weighted gene co-expression network analysis is a systems biology method for describing the correlation patterns among genes across microarray samples. Weighted correlation network analysis (WGCNA) can be used for finding clusters (modules) of highly correlated genes, for summarizing such clusters using the module eigengene or an intramodular hub gene, for relating modules to one another and to external sample traits (using eigengene network methodology), and for calculating module membership measures. Correlation networks facilitate network based gene screening methods that can be used to identify candidate biomarkers or therapeutic targets. These methods have been successfully applied in various biological contexts, e.g. cancer, mouse genetics, yeast genetics, and analysis of brain imaging data. While parts of the correlation network methodology have been described in separate publications, there is a need to provide a user-friendly, comprehensive, and consistent software implementation and an accompanying tutorial.

**Results:**

The WGCNA R software package is a comprehensive collection of R functions for performing various aspects of weighted correlation network analysis. The package includes functions for network construction, module detection, gene selection, calculations of topological properties, data simulation, visualization, and interfacing with external software. Along with the R package we also present R software tutorials. While the methods development was motivated by gene expression data, the underlying data mining approach can be applied to a variety of different settings.

**Conclusion:**

The WGCNA package provides R functions for weighted correlation network analysis, e.g. co-expression network analysis of gene expression data. The R package along with its source code and additional material are freely available at .

## Background

Correlation networks are increasingly being used in biology to analyze large, high-dimensional data sets. Correlation networks are constructed on the basis of correlations between quantitative measurements that can be described by an *n *× *m *matrix *X *= [*x*_*il*_] where the row indices correspond to network nodes (*i *= 1, . . ., *n*) and the column indices (*l *= 1, . . ., *m*) correspond to sample measurements:

(1) $$X=[x_{ij}]=\left(\begin{array}{c} x_{1}\\ x_{2}\\ \cdots \\ x_{n} \end{array}\right)$$

We refer to the *i*-th row *x*_*i *_as the *i*-th *node profile *across *m *sample measurements.

Sometimes a quantitative measure (referred to as *sample trait*) is provided for the columns of *X*. For example, *T *= (*T*_1_, . . ., *T*_*m*_) could measure survival time or it could be a binary indicator variable (disease status). Abstractly speaking, we define a sample trait *T *as a vector with *m *components that correspond to the columns of the data matrix *X*. A sample trait can be used to define a node significance measure. For example, a *trait-based node significance measure *can be defined as the absolute value of the correlation between the *i*-th node profile *x*_*i *_and the sample trait *T*:

(2) *GS*_*i *_= |*cor*(*x*_*i*_, *T*)|.

Alternatively, a correlation test p-value [1] or a regression-based p-value for assessing the statistical significance between *x*_*i *_and the sample trait *T *can be used to define a p-value based node significance measure, for example by defining

(3) *GS*_*i *_= -log *p*_*i*_.

The rationale behind correlation network methodology is to use network language to describe the pairwise relationships (correlations) between the rows of *X *(Equation 1). Although other statistical techniques exist for analyzing correlation matrices, network language is particularly intuitive to biologists and allows for simple social network analogies. Correlation networks can be used to address many analysis goals including the following. First, correlation networks can be used to find clusters (modules) of interconnected nodes. Thus, a network module is a set of rows of *X *(Equation 1) which are closely connected according to a suitably defined measure of interconnectedness.

A second analysis goal is to summarize the node profiles of a given module by a representative, e.g. a highly connected hub node, which is centrally located in the module. Focusing the analysis on module or their representatives amounts to a network-based data reduction method. Relating modules instead of nodes to a sample trait can alleviate the multiple testing problem.

A third analysis goal is to identify 'significant' modules. Toward this end, a node significance measure can be used to identify modules with high average node significance (referred to as module significance).

A fourth analysis goal is to annotate all network nodes with respect to how close they are to the identified modules. This can be accomplished by defining a fuzzy measure of module memberships that generalizes the binary module membership indicator to a quantitative measure. Fuzzy measures of module membership can be used to identify nodes that lie intermediate between and close to two or more modules.

A fifth analysis goal is to define the network neighborhood of a given seed set of nodes. Intuitively speaking, a neighborhood is composed of nodes that are highly connected to a given set of nodes. Thus, neighborhood analysis facilitates a guilt-by-association screening strategy for finding nodes that interact with a given set of interesting nodes.

A sixth analysis goal is to screen for nodes based on node screening criteria which can be based on a node significance measure, on module membership information, on network topological properties (e.g. high connectivity), etc.

A seventh analysis goal is to contrast one network with another network. This differential network analysis can be used to identify changes in connectivity patterns or module structure between different conditions. An eighth analysis goal is to find shared modules between two or more networks (consensus module analysis). Since by definition consensus modules are building blocks in multiple networks, they may represent fundamental structural properties of the network.

The above incomplete enumeration of analysis goals shows that correlation networks can be used as a data exploratory technique (similar to cluster analysis, factor analysis, or other dimensional reduction techniques) and as a screening method. For example, correlation networks can be used to screen for modules and intramodular hubs that relate to a sample trait. Correlation networks allow one to generate testable hypotheses that should be validated in independent data or in designed validation experiments.

### Gene Co-Expression Networks

In the following, we focus on gene co-expression networks which represent a major application of correlation network methodology. Co-expression networks have been found useful for describing the pairwise relationships among gene transcripts [2-9]. In co-expression networks, we refer to nodes as 'genes', to the node profile *x*_*i *_as the gene expression profile, and to the node significance measure *GS*_*i *_as the gene significance measure. A glossary of important network-related terms can be found in Table 1. Here we introduce an R software package that summarizes and extends our earlier work on weighted gene co-expression network analysis (WGCNA) [5,10-12]. WGCNA has been used to analyze gene expression data from brain cancer [10], yeast cell cycle [13], mouse genetics [14-17], primate brain tissue [18-20], diabetes [21], chronic fatigue patients [22] and plants [23]. While these publications have made R software code available in various forms, there is a need for a comprehensive R package that summarizes and standardizes methods and functions. To address this need, we introduce the WGCNA R package which also includes enhanced and novel functions for co-expression network analysis.

**Table 1:** Glossary of WGCNA Terminology.

Term	Definition
Co-expression network	We define co-expression networks as undirected, weighted gene networks. The nodes of such a network correspond to gene expression profiles, and edges between genes are determined by the pairwise correlations between gene expressions. By raising the absolute value of the correlation to a power *β *≥ 1 (soft thresholding), the weighted gene co-expression network construction emphasizes high correlations at the expense of low correlations. Specifically, *a*_*ij *_= |cor(*x*_*i*_, *x*_*j*_)|^*β *^represents the adjacency of an unsigned network. Optionally, the user can also specify a signed co-expression network where the adjacency is defined as *a*_*ij *_= |(1 + cor(*x*_*i*_, *x*_*j*_))*/*2|^*β*^.

Module	Modules are clusters of highly interconnected genes. In an unsigned co-expression network, modules correspond to clusters of genes with high absolute correlations. In a signed network, modules correspond to positively correlated genes.

Connectivity	For each gene, the connectivity (also known as degree) is defined as the sum of connection strengths with the other network genes: *k*_*i *_= ∑_*u*≠*i*_*a*_*ui*_. In co-expression networks, the connectivity measures how correlated a gene is with all other network genes.

Intramodular connectivity *k*_*IM*_	Intramodular connectivity measures how connected, or co-expressed, a given gene is with respect to the genes of a particular module. The intramodular connectivity may be interpreted as a measure of module membership.

Module eigengene *E*	The module eigengene *E *is defined as the first principal component of a given module. It can be considered a representative of the gene expression profiles in a module.

Eigengene significance	When a microarray sample trait *y *is available (e.g. case control status or body weight), one can correlate the module eigengenes with this outcome. The correlation coefficient is referred to as eigengene significance.

Module Membership, also known as eigengene-based connectivity *k*_*ME*_	For each gene, we define a "fuzzy" measure of module membership by correlating its gene expression profile with the module eigengene of a given module. For example, *MM*^*blue*^(*i*) = $K_{cor,i}^{blue}$ = cor(*x*_*i*_, *E*^*blue*^) measures how correlated gene *i *is to the blue module eigengene. *MM*^*blue*^(*i*) measures the membership of the *i*-th gene with respect to the blue module. If *MM*^*blue*^(*i*) is close to 0, the *i*-th gene is not part of the blue module. On the other hand, if *MM*^*blue*^(*i*) is close to 1 or -1, it is highly connected to the blue module genes. The sign of module membership encodes whether the gene has a positive or a negative relationship with the blue module eigengene. The module membership measure can be defined for all input genes (irrespective of their original module membership). It turns out that the module membership measure is highly related to the intramodular connectivity *k*_*IM*_. Highly connected intramodular hub genes tend to have high module membership values to the respective module.

Hub gene	This loosely defined term is used as an abbreviation of "highly connected gene." By definition, genes inside co-expression modules tend to have high connectivity.

Gene significance *GS*	To incorporate external information into the co-expression network, we make use of gene significance measures. Abstractly speaking, the higher the absolute value of *GS*_*i*_, the more biologically significant is the *i*-th gene. For example, *GS*_*i *_could encode pathway membership (e.g. 1 if the gene is a known apoptosis gene and 0 otherwise), knockout essentiality, or the correlation with an external microarray sample trait. A gene significance measure could also be defined by minus log of a p-value. The only requirement is that gene significance of 0 indicates that the gene is not significant with regard to the biological question of interest. The gene significance can take on positive or negative values.

Module significance	Module significance is determined as the average absolute gene significance measure for all genes in a given module. When gene significance is defined as the correlation of gene expression profiles with an external trait *y*, this measure tends to be highly related to the correlation between the module eigengene and *y*.

## Results

**Figure 1:**
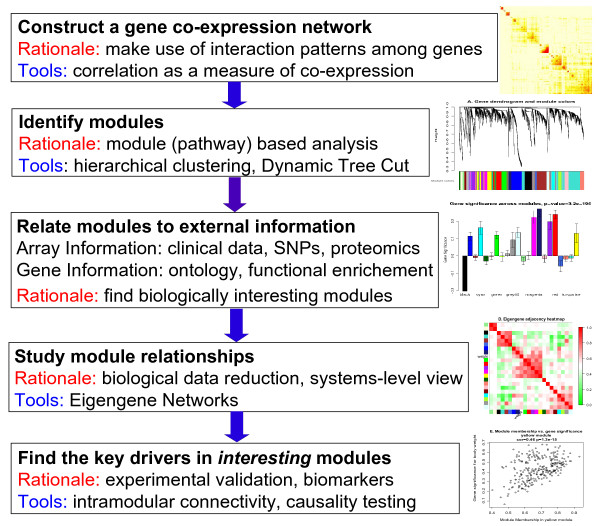
**Overview of WGCNA methodology**. This flowchart presents a brief overview of the main steps of Weighted Gene Co-expression Network Analysis.

Figure 1 provides an overview of typical analysis steps and the rationale behind them. To determine whether a co-expression module is biologically meaningful, one can use functional enrichment and gene ontology information.

### Overview of functions included in the WGCNA package

The WGCNA package contains a comprehensive set of functions for performing a correlation network analysis of large, high-dimensional data sets. Functions in the WGCNA package can be divided into the following categories: 1. network construction; 2. module detection; 3. module and gene selection; 4. calculations of topological properties; 5. data simulation; 6. visualization; 7. interfacing with external software packages. An exhaustive list of implemented functions together with detailed descriptions is provided in the R package manual posted on our web site. Here we briefly outline the main functionality of the package and highlight new contributions.

### Category 1: Functions for network construction

A network is fully specified by its *adjacency matrix a*_*ij*_, a symmetric *n *× *n *matrix with entries in [0, 1] whose component *a*_*ij *_encodes the network connection strength between nodes *i *and *j*. To calculate the adjacency matrix, an intermediate quantity called the *co-expression similarity s*_*ij *_is first defined. The default method defines the co-expression similarity *s*_*ij *_as the absolute value of the correlation coefficient between the profiles of nodes *i *and *j*:

*s*_*ij *_= |*cor*(*x*_*i*_, *x*_*j*_)|.

The WGCNA package also implements alternative co-expression measures, e.g. more robust measures of correlation (the biweight midcorrelation [24] or the Spearman correlation). A signed co-expression measure can be defined to keep track of the sign of the co-expression information. For convenience, we define the co-expression similarity measure such that it takes on values in [0, 1].

**Figure 2:**
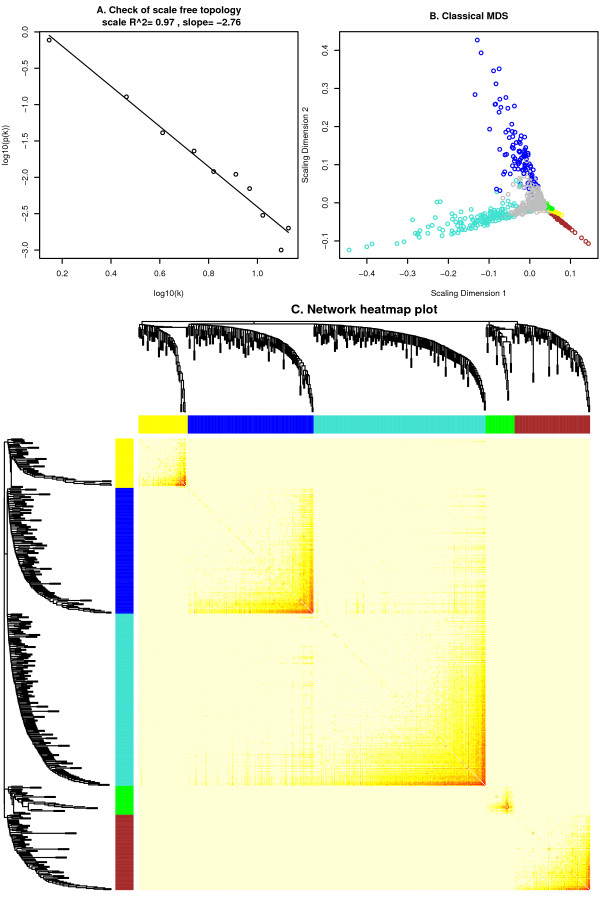
**Network visualization plots**. A. Log-log plot of whole-network connectivity distribution. The *x*-axis shows the logarithm of whole network connectivity, *y*-axis the logarithm of the corresponding frequency distribution. On this plot the distribution approximately follows a straight line, which is referred to as approximately scale-free topology. B. Results of classical multidimensional scaling. Modules tend to form separate 'fingers' in this plot. Intramodular hub genes are located at the finger tips. C. Network heatmap plot. Branches in the hierarchical clustering dendrograms correspond to modules. Color-coded module membership is displayed in the color bars below and to the right of the dendrograms. In the heatmap, high co-expression interconnectedness is indicated by progressively more saturated yellow and red colors. Modules correspond to blocks of highly interconnected genes. Genes with high intramodular connectivity are located at the tip of the module branches since they display the highest interconnectedness with the rest of the genes in the module.

Using a thresholding procedure, the co-expression similarity is transformed into the adjacency. An unweighted network adjacency *a*_*ij *_between gene expression profiles *x*_*i *_and *x*_*j *_can be defined by hard thresholding the co-expression similarity *s*_*ij *_as

(4) $$a_{ij}=\{\begin{array}{ll} 1 & \text{if }s_{ij}\geq \tau ;\\ 0 & \text{otherwise}, \end{array}$$

where *τ *is the "hard" threshold parameter. Thus, two genes are linked (*a*_*ij *_= 1) if the absolute correlation between their expression profiles exceeds the (hard) threshold *τ*. The hard-thresholding procedure is implemented in the function signumAdjacencyFunction. While unweighted networks are widely used, they do not reflect the continuous nature of the underlying co-expression information and may thus lead to an information loss. In contrast, weighted networks allow the adjacency to take on continuous values between 0 and 1. A weighed network adjacency can be defined by raising the co-expression similarity to a power [5,10]:

(5) $$a_{ij}=s_{ij}^{\beta },$$

with *β *≥ 1. The function adjacency calculates the adjacency matrix from expression data. The adjacency in Equation 5 implies that the weighted adjacency *a*_*ij *_between two genes is proportional to their similarity on a logarithmic scale, *log*(*a*_*ij*_) = *β *× *log*(*s*_*ij*_). Adjacency functions for both weighted and unweighted networks require the user to choose threshold parameters, for example by applying the approximate scale-free topology criterion [5]. The package provides functions pickSoftThreshold, pickHardThreshold that assist in choosing the parameters, as well as the function scaleFreePlot for evaluating whether the network exhibits a scale free topology. Figure 2A shows a plot identifying scale free topology in simulated expression data.

### Category 2: Functions for module detection

Once the network has been constructed, module detection is often a logical next step. Modules are defined as clusters of densely interconnected genes. Several measures of network interconnectedness are described in [25]. As default, we we use the topological overlap measure [5,25-27] since it has worked well in several applications. WGCNA identifies gene modules using unsupervised clustering, i.e. without the use of a priori defined gene sets. The user has a choice of several module detection methods. The default method is hierarchical clustering using the standard R function hclust [28]; branches of the hierarchical clustering dendrogram correspond to modules and can be identified using one of a number of available branch cutting methods, for example the constant-height cut or two Dynamic Branch Cut methods [29].

In Figure 2C we show a network heatmap plot (interconnectivity plot) of a gene network together with the corresponding hierarchical clustering dendrograms and the resulting modules. Figure 2B provides an alternate visualization of the module structure via a multi-dimensional scaling plot (standard R function cmdscale).

One drawback of hierarchical clustering is that it can be difficult to determine how many (if any) clusters are present in the data set. Although the height and shape parameters of the Dynamic Tree Cut method provide improved exibility for branch cutting and module detection, it remains an open research question how to choose optimal cutting parameters or how to estimate the number of clusters in the data set [30]. While our default parameter values have worked well in several applications, in practice we recommend to carry out a cluster stability/robustness analysis. A co-expression module may reflect a true biological signal (e.g. a pathway) or it may reflect noise (e.g. a technical artifacts, tissue contamination, or a false positive). To test whether the identified modules are biologically meaningful, gene ontology information (functional enrichment analysis) can be used. Toward this end, we provide an R tutorial that describes how to interface the WGCNA package with relevant external software packages and databases.

#### Summarizing the profiles of a module

Several options have been implemented for summarizing the gene expression profiles of a given module. For example, the function moduleEigengenes represents the module expressions of the *q*-th module by the module eigengene *E*^(*q*)^, defined as the first principal component of the expression matrix. The eigengene *E *can be thought of as a weighted average expression profile. Eigengene calculation incorporates imputation of missing values implemented in the package impute [31,32]. Alternatively, the user can use the intramodular connectivity measure to define the most highly connected intramodular hub gene as the module representative. One can show that intramodular hub genes are highly correlated with the module eigengene [11].

#### Fuzzy measure of module membership

Hierarchical clustering and most other standard clustering methods such as Partitioning Around Medoids (PAM) [28] result in a binary module assignment, i.e. a node is either in or outside of a module. In some applications it may be advantageous to define a continuous, fuzzy measure of module membership for all nodes. Such measure is particularly useful to identify nodes that lie near the boundary of a module, or nodes that are intermediate between two or more modules. As explained in detail in [11], the module membership of node *i *in module *q *can be defined as

(6) $$K_{cor,i}^{\left(q\right)}:=\text{cor}(x_{i},E^{\left(q\right)}),$$

where *x*_*i *_is the profile of node *i *and *E*^(*q*) ^is the module eigengene of module *q*. The module membership measure $K_{cor,i}^{\left(q\right)}$ lies in [-1, 1] and specifies how close node *i *is to module *q*, *q *= 1, . . ., *Q*. The larger |$K_{cor,i}^{\left(q\right)}$|, the more similar node *i *is to the eigengene of the *q*-th module. In some publications [14,15], $K_{cor,i}^{\left(q\right)}$ is referred to as signed module eigengene (ME) based connectivity measure *K*_*ME*_. This is the reason why we named the corresponding R function signedKME.

#### Automatic block-wise module detection

Many microarray gene expression measurements report expression levels of tens of thousands of distinct genes (or probes). Building and analyzing a full network among such a large number of nodes can be computationally challenging because of memory size and processor speed limitations. The WGCNA package contains several improvements that address this challenge. The function blockwiseModules is designed to handle network construction and module detection in large data sets. The function first pre-clusters nodes into large clusters, referred to as blocks, using a variant of k-means clustering (function projectiveKMeans). Next, hierarchical clustering is applied to each block and modules are defined as branches of the resulting dendrogram. To synthesize the module detection results across blocks, an automatic module merging step (function mergeCloseModules) is performed that merges modules whose eigengenes are highly correlated. The time and memory savings of the block-wise approach are substantial: a standard, single-block network analysis of *n *nodes requires *O*(*n*^2^) memory and *O*(*n*^3^) calculations, while the block-wise approach with block size *n*_*b *_requires only *O*($n_{b}^{2}$) memory and *O*(*n*$n_{b}^{2}$) calculations, making an analysis of say 50 000 genes in blocks of 7 000 feasible on a standard computer.

#### Consensus module detection

When dealing with multiple adjacency matrices representing different networks, it can be interesting to find *consensus modules*, defined as modules that are present in all or most networks [12]. Intuitively, two nodes should be connected in a consensus network only if all of the input networks agree on that connection. This naturally suggest to define the consensus network similarity between two nodes as the minimum of the input network similarities. In certain cases it may be useful to replace minimum by a suitable quantile (e.g. the first quartile) since the resulting measure may be more robust. Consensus module detection can be performed step-by-step for maximum control and exibility, or in one step using the function blockwiseConsensusModule that calculates consensus modules across given data sets in a block-wise manner analogous to the block-wise module detection in a single data set.

### Category 3: Functions for module and gene selection

**Figure 3:**
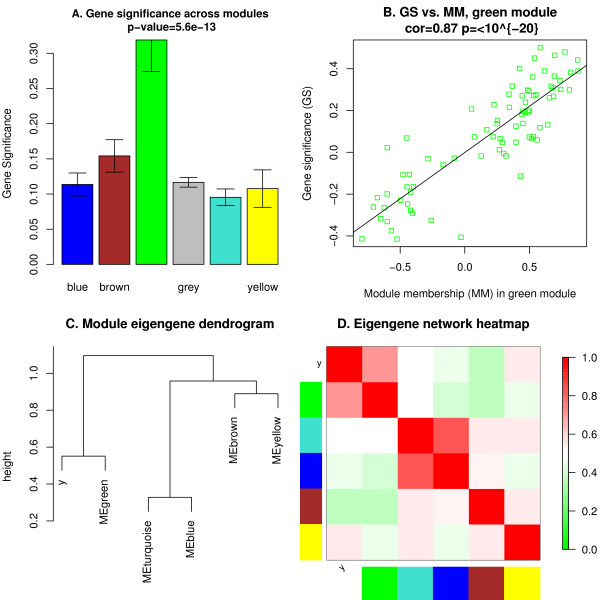
**Module and eigengene network plots**. A. Barplot of mean gene significance across modules. In this example we use a trait-based gene significance, Equation 2. The higher the mean gene significance in a module, the more significantly related the module is to the clinical trait of interest. B. Scatterplot of gene significance (*y*-axis) vs. module membership (*x*-axis) in the most significant module (green module, see panel A). In modules related to a trait of interest, genes with high module membership often also have high gene significance. C. Hierarchical clustering dendrogram of module eigengenes (labeled by their colors) and the microarray sample trait *y*. D. Heatmap plot of the adjacencies in the eigengene network including the trait *y*. Each row and column in the heatmap corresponds to one module eigengene (labeled by color) or the trait (labeled by *y*). In the heatmap, green color represents low adjacency (negative correlation), while red represents high adjacency (positive correlation).

Finding biologically or clinically significant modules and genes is a major goal of many co-expression analyses. The definition of biological or clinical significance depends on the research question under consideration. Abstractly speaking, we define a gene significance measure as a function *GS *that assigns a non-negative number to each gene; the higher *GS*_*i *_the more *biologically *significant is gene *i*. In functional enrichment analysis, a gene significance measure could indicate pathway membership. In gene knock-out experiments, gene significance could indicate knock-out essentiality. A microarray sample trait *T *can be used to define a trait-based gene significance measure as the absolute correlation between the trait and the expression profiles, Equation 2. A measure of module significance can be defined as average gene significance across the module genes (Figure 3A). When dealing with a sample trait *T*, a measure of statistical significance between the module eigengene *E *and the trait *T *can be defined, for example, using correlation (Equation 2) or a p-value (Equation 3) obtained from a univariate regression model between *E *and *T*. Modules with high trait significance may represent pathways associated with the sample trait. Genes with high module membership in modules related to traits (Figure 3B) are natural candidates for further validation [10,14,15,18].

### Category 4: Functions for studying topological properties

Many topological properties of networks can be succinctly described using network concepts, also known as network statistics or indices [11,33]. Network concepts include whole network connectivity (degree), intramodular connectivity, topological overlap, the clustering coefficient, density etc. Differential analysis of network concepts such as intramodular connectivity may reveal regulatory changes in gene expressions [15,18]. The WGCNA package implements several functions, such as softConnectivity, intramodularConnectivity, TOMSimilarity, clusterCoef, networkConcepts, for computing these network concepts. Basic R functions can be used to create summary statistics of these concepts and for testing their differences across networks.

#### Network concepts for measuring cluster structure

**Figure 4:**
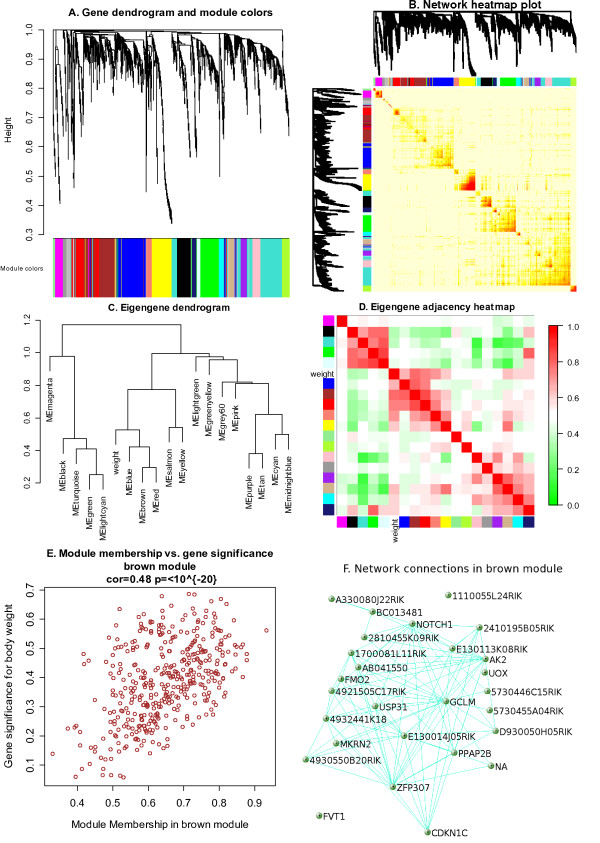
**Example WGCNA analysis of liver expression data in female mice**. A. Gene dendrogram obtained by average linkage hierarchical clustering. The color row underneath the dendrogram shows the module assignment determined by the Dynamic Tree Cut. B. Heatmap plot of topological overlap in the gene network. In the heatmap, each row and column corresponds to a gene, light color denotes low topological overlap, and progressively darker red denotes higher topological overlap. Darker squares along the diagonal correspond to modules. The gene dendrogram and module assignment are shown along the left and top. C. Hierarchical clustering of module eigengenes that summarize the modules found in the clustering analysis. Branches of the dendrogram (the meta-modules) group together eigengenes that are positively correlated. D. Heatmap plot of the adjacencies in the eigengene network including the trait weight. Each row and column in the heatmap corresponds to one module eigengene (labeled by color) or weight. In the heatmap, green color represents low adjacency (negative correlation), while red represents high adjacency (positive correlation). Squares of red color along the diagonal are the meta-modules. E. A scatterplot of gene significance for weight (GS, Equation 2) versus module membership (MM, Equation 6) in the brown module. GS and MM exhibit a very significant correlation, implying that hub genes of the brown module also tend to be highly correlated with weight. F. The network of the 30 most highly connected genes in the brown module. In this network we only display a connection of the corresponding topological overlap is above a threshold of 0.08.

Gene clustering trees and TOM plots that visualize interconnectivity patterns often suggest the presence of large modules. Network theory offers a wealth of intuitive network concepts for describing the pairwise relationships among genes that are depicted in cluster trees and heat maps [11]. To illustrate this point, we describe two network concepts in the following. By visual inspection of Figures 2C and 4B, genes appear to be highly interconnected, e.g. turquoise module genes form a reddish square in the TOM plot. This property of dense connections among the genes of module *q *can be measured using the concept of module density, which is defined as the average adjacency of the module genes:

(7) $$Density(A^{\left(q\right)})=\frac{\sum_{i}\sum_{j\neq i}a_{ij}^{\left(q\right)}}{n^{\left(q\right)}(n^{\left(q\right)}-1)}$$

where *A*^(*q*) ^denotes the *n*^(*q*) ^× *n*^(*q*) ^adjacency matrix corresponding to the sub-network formed by the genes of module *q*. Another useful concept is the *clustering coefficient *of gene *i*, which is a measure of 'cliquishness' [34]. Specifically,

(8) $$ClusterCoef_{i}=\frac{\sum_{l\neq i}\sum_{m\neq i,l}a_{il}a_{lm}a_{mi}}{\left\{{\left(\sum_{l\neq i}a_{il}\right)}^{2}-\sum_{l\neq i}{\left(a_{il}\right)}^{2}\right\}}.$$

In unweighted networks, *ClusterCoef*_*i *_equals 1 if and only if all neighbors of gene *i *are also linked to each other. For weighted networks, 0 ≤ *a*_*ij *_≤ 1 implies that 0 ≤ *ClusterCoef*_*i *_≤ 1 [5]. The mean clustering coefficient has been used to measure the extent of module structure present in a network [26,34].

### Category 5: Functions for simulating microarray data with modular structure

Simple yet sufficiently realistic simulated data is often important for evaluation of novel data mining methods. The WGCNA package includes simulation functions simulateDatExpr, simulateMultiExpr, simulateDatExpr5Modules that result in expression data sets with a customizable modular (cluster) structure. The user can choose the modular structure by specifying a set of seed eigengenes, one for each module, around which each module is built. Module genes are simulated to exhibit progressively lower correlations with the seed which leads to genes with progressively lower intramodular connectivity. The user can specify module sizes and the number of background genes, i.e. genes outside of the modules. The seed eigengenes can be simulated to reflect dependence relationships between the modules (function simulateEigengeneNetwork).

### Category 6: Visualization functions

Module structure and network connections in the expression data can be visualized in several different ways. For example, the co-expression module structure can be visualized by heatmap plots of gene-gene connectivity that can be produced using the function TOMplot. Examples are presented in Figures 2C and 4B. An alternative is a multi-dimensional scaling plot; an example is presented in Figure 2B. Relationships among modules can be summarized by a hierarchical clustering dendrogram of their eigengenes, or by a heatmap plot of the corresponding eigengene network (function labeledHeatmap), illustrated in Figures 3C, D, and 4C, D. The package includes several additional functions designed to aid the user in visualizing input data and results. These functions rely on basic plotting functions provided in R and the packages sma [35] and fields [36].

### Category 7: Functions for interfacing with other software packages

To enhance the integration of WGCNA results with other network visualization packages and gene ontology analysis software, we have created several R functions and corresponding tutorials. For example, our R functions exportNetworkToVisANT and exportNetworkToCytoscape allow the user to export networks in a format suitable for VisANT [37] and Cytoscape [38], respectively.

Our online R tutorials also show how to interface WGCNA results with gene ontology packages available directly in R, e.g. GOSim [39]. Many gene ontology based functional enrichment analysis software programs such as David [40], AmiGO [41], Webgestalt [42] simply take lists of gene identifiers as input. Ingenuity Pathway Analysis allows the user to input gene expression data or gene identifiers.

### Mouse Data Application

As an example of the type of analysis one can perform with WGCNA, we describe a network analysis of liver expression data from female mice. The data and biological findings of this analysis have been described in [14]. Briefly, mRNA levels in female mouse livers were measured by microarrays with over 23,000 probe sets. In addition to the expression data, multiple physiological and metabolic traits were measured. For computational reasons, the original analysis presented in [14] was restricted to 3600 most connected genes, and for simplicity we will work with the same set of genes (although we note that the presented package is capable of handling all genes as well). While we do use the same data, the module detection methods are slightly different and the results are similar but not the same. The code used to perform this analysis is part of the tutorials posted on our webpage.

The network and the 18 identified modules are depicted in Figures 4A, B. To understand the physiologic significance of the modules, we correlated the 18 module eigengenes with physiological traits such as body weight, cholesterol level, insulin level. The full module-trait correlation table is presented in the accompanying tutorial.

In the following, we will only consider mouse body weight as sample trait. The module eigengenes of the following three modules were highly correlated with body weight: brown (409 genes, correlation with weight *r *= 0.59, correlation p-value *p *= 5 × 10^-14^), red (221 genes, *r *= 0.51, *p *= 3 × 10^-10^), and salmon (91 genes, *r *= 0.43, *p *= 2 × 10^-7^).

We used the online software David [40] to determine whether the three body weight related modules were significantly enriched with regard to known gene ontologies. The brown module is significantly enriched in categories "glycoprotein" (*p *= 2 × 10^-24^, Benjamini corrected) and "signal" (*p *= 1 × 10^-22^). The red module is enriched in "cell cycle" (*p *= 9 × 10^-24^) and "chromosome" (*p *= 5 × 10^-20^). The salmon module is most significantly enriched in the category "lipid synthesis" (*p *= 1 × 10^-16^). Overall, the high enrichment scores suggest that these modules are indeed biologically meaningful.

To study the relationships between modules, we correlate their eigengenes. In general, relationships between modules can be studied by using correlation networks between eigengenes (i.e. the nodes correspond to eigengenes). In these meta-networks between modules, the adjacency between modules reflects the correlation between the module eigengenes, and modules of eigengenes are referred to as meta-modules [12]. A sample trait such as body weight can be incorporated as an additional node of the eigengene network. The adjacency between the sample trait and an eigengene is sometimes referred to as the eigengene significance [11]. Figures 4C, D depict the eigengene network using a dendrogram (hierarchical cluster tree) and a heatmap plot. We find that eigengenes may exhibit highly significant correlations, e.g. the red and brown modules are highly correlated. Groups of correlated eigengenes corresponds to meta-modules and are recognizable as branches of the eigengene dendrogram, and as reddish squares along the diagonal of the heatmap plot. Figures 4C indicates that there are four meta-modules (branches). Body weight falls within the meta-module grouping together the blue, brown, red, salmon, and yellow modules. In practice, it is difficult to determine whether the modules underlying a meta-module are truly distinct or whether they should be merged. Sometimes gene ontology information can provide some clues.

It is interesting to find centrally located intramodular hub genes in the body weight related modules since their expression profile represents that of the entire module [11]. To find intramodular hub genes, one can use the module membership measure *K*, Equation 6. Figure 4E shows a scatterplot between the body weight based gene significance measure *GS*_*i*_, Equation 2 and module membership in the brown module.

The high correlation between gene significance and module membership implies that hubgenes in the brown module also tend to be highly correlated with body weight. This suggests that both gene significance and module membership (intramodular connectivity) can be combined in a systems biologic screening method for finding body weight related genes [15]. Figure 4F shows a Visant plot among the most connected genes in the brown module. This brief description illustrates how WGCNA can lead to testable hypotheses that require validation in independent data sets. A tutorial underlying this example and Figure 4 can be found on our webpage.

### Tutorials

We provide a comprehensive set of online tutorials that guide the user through major steps of correlation network analysis. The tutorials provide R code the user can copy-and-paste into an R session, along with comments and explanations of both the input and output. The code is organized into short sections, each of which addresses a particular task. In particular, the tutorials cover the following topics: correlation network construction, step-by-step and automatic module detection, consensus module detection, eigengene network analysis, differential network analysis, interfacing with external software packages, and data simulation. The tutorials use both simulated and real gene expression data sets.

## Discussion

The WGCNA package complements other network related packages for R, such as the general network structures in Bioconductor [6], gene network enrichment analysis [43], functional analysis of gene co-expression networks [44], and others. While most of the existing packages focus only on unweighted networks, WGCNA implements methods for both weighted and unweighted correlation networks. WGCNA can be used as a data exploratory tool or as a gene screening (ranking) method. For example, WGCNA can be used to explore the module (cluster) structure in a network, to measure the relationships between genes and modules (module membership information), to explore the relationships among modules (eigengene networks), and to rank-order genes or modules (e.g. with regard to their relationship with a sample trait). WGCNA can be used to generate testable hypotheses for validation in independent data sets. For example, WGCNA may suggest that a module (e.g. a putative pathway) is associated with a disease outcome. Since correlation networks are based on correlations between quantitative variables, one can use a correlation test p-value [1] or a regression-based p-value for assessing the statistical significance between pairs of variables. For example, it is straightforward to attach a significance level to the fuzzy module membership measures $K_{cor,i}^{\left(q\right)}$. The relationship between standard microarray data mining techniques and gene co-expression network analysis is discussed in [11].

Users should be aware of the limitations of the methods implemented in the WGCNA package. First, WGCNA assumes that the microarray data have been properly pre-processed and normalized. To normalize the expression data, several R functions have been implemented in the Bioconductor packages [45]. Although all normalization methods are mathematically compatible with WGCNA, we recommend to use the biologically most meaningful normalization method with respect to the application under consideration. Second, similar to most other data mining methods, the results of WGCNA can be biased or invalid when dealing with technical artefacts, tissue contaminations, or poor experimental design. Third, although several co-expression module detection methods are implemented, the package does not provide means to determine which method is best. While the default hierarchical clustering methods have performed well in several real data applications, it would be desirable to compare these and other methods on multiple real benchmark data sets. Fourth, this package is limited to undirected networks. Methods for orienting edges and constructing directed networks have been presented in the literature, for example in [46-48].

## Conclusion

The WGCNA R package provides a comprehensive set of functions for performing weighted correlation network analysis. The WGCNA package can also be used to describe the correlation structure between gene expression profiles, image data, genetic marker data, proteomics data, and other high-dimensional data.

## Availability and requirements

Project name: WGCNA R package

Project home page: 

Operating system(s): Platform independent

Programming language: R

Licence: GNU GPL 3

## Authors' contributions

Both authors jointly developed the methods and wrote the article. PL packaged the functions into an R package. Both authors read and approved the final manuscript.

## Acknowledgements

We would like to thank Jun Dong, Tova Fuller, Dan Geschwind, Winden Kellen, Wen Lin, Jake Lusis, Mike Mason, Jeremy Miller, Paul Mischel, Stan Nelson, Mike Oldham, Angela Presson, Atila Van Nas, and Lin Wang for helpful discussions and suggestions. The work was supported in part by grants P50CA092131, 5P30CA016042-28, and NS050151-01.
